# Down-regulated lncRNA SBF2-AS1 inhibits tumorigenesis and progression of breast cancer by sponging microRNA-143 and repressing RRS1

**DOI:** 10.1186/s13046-020-1520-5

**Published:** 2020-01-17

**Authors:** Wenfei Xia, Yun Liu, Teng Cheng, Tao Xu, Menglu Dong, Xiaopeng Hu

**Affiliations:** 1 Department of Breast and Thyroid surgery, Division of General Surgery, Tongji Hospital, Tongji Medical College, Huazhong University of Science and Technology, No. 1095, Jiefang Avenue, Qiaokou District, Wuhan City, Hubei Province 430030 People’s Republic of China; 2 Department of ENT, Tongji Hospital, Tongji Medical College, Huazhong University of Science and Technology, Wuhan City, Hubei Province 430030 People’s Republic of China

**Keywords:** Breast cancer, Long non-coding RNA SBF2-AS1, MicroRNA-143, Resistance to ralstonia solanacearum 1, Tumor growth, Invasion, Migration, Apoptosis

## Abstract

**Background:**

Recently, the roles of long non-coding RNAs (lncRNAs) and microRNAs (miRNAs) in human diseases have been unveiled, this research was conducted to explore the impacts of lncRNA SET-binding factor 2-antisense RNA1 (SBF2-AS1), miR-143 and resistance to ralstonia solanacearum 1 (RRS1) on breast cancer (BC) development.

**Methods:**

The expression of SBF2-AS1, miR-143 and RRS1 in BC tissues, as well as in MDA-MB-231 and MCF-7 cell lines were assessed. Subsequently, the cells were transfected with miR-143 mimics or/and silenced or overexpressed SBF2-AS1 plasmids, and their negative controls. Then the proliferation, colony formation ability, cell cycle arrest, apoptosis, invasion and migration of the cells were assessed through gain- and loss-of-function experiments. Furthermore, the tumor growth, ki-67 expression and apoptosis in vivo were observed by subcutaneous tumorigenesis in nude mice. Binding relation between SBF2-AS1 and miR-143, and that between miR-143 and RRS1 were confirmed.

**Results:**

SBF2-AS1 and RRS1 were amplified, while miR-143 was reduced in BC tissues and cells. Reduced SBF2-AS1 and elevated miR-143 could repress the proliferation, invasion and migration via restraining RRS1 expression. Moreover, knockdown of SBF2-AS1 up-regulated miR-143 to promote the apoptosis of BC cells by downregulating RRS1, resulting in a prohibitive effect on the tumorigenesis and progression of BC. Results of in vivo experiments indicated that the inhibited SBF2-AS1 and overexpressed miR-143 could restrict BC cell proliferation and promote apoptosis, and decelerate tumor growth in xenografts.

**Conclusion:**

We have discovered in this study that down-regulated SBF2-AS1 could inhibit tumorigenesis and progression of BC by up-regulation miR-143 and repressing RRS1, which provides basic therapeutic considerations for a novel target against BC.

## Background

Breast cancer (BC) is a kind of cancer that specifically occurs in mammary epithelial tissues, and is the commonest cancer in women all over the world with a morbidity of 25.1% among all the cancers, causing breast cancer the 2nd contributory factor of cancer-related death after lung cancer in the world [1]. The mortality of BC has been reduced in recent years in most high-income countries owing to the developed therapy and earlier diagnosis. Nevertheless, an elevating mortality was still existed in certain countries [2]. The factors including gender, age, obesity, alcohol consumption, oral contraceptive, hormone replacement treatment, hereditary tendency and family history are all demonstrated to be implicated in the BC tumorigenesis [3]. Moreover, some clinicopathological parameters including histological type, histological grade, lymph node metastasis (LNM), and clinical stages have been generally applied in the management of BC, yet some patients with the same clinicopathological features have distinct prognosis [4]. In order to promote the therapeutic efficiency and prognosis of BC, critical mechanisms that modulate the tumor growth and progression of BC are sorely needed.

Long non-coding RNAs (lncRNAs) have been affirmed in human cancers and were found to exert critical functions via interacting with DNA, RNA, protein molecules and their combinations [5]. LncRNA SBF2 antisense RNA 1 (SBF2-AS1) is one of the lncRNAs that situated at human chromosome 11p15.1 and contains 2708 nucleotides (nt) [6]. The modulatory impacts of SBF2-AS1 have been verified in several kinds of human diseases, for example, Chen et al. have discovered that SBF2-AS1 was related to the procedure of early-stage lung adenocarcinoma [7]. More than that, the promotive role of SBF2-AS1 in the procedures of cervical cancer has also been clarified in a recent research [8], while the function mechanisms of SBF2-AS1 in BC have not been illustrated yet. Furthermore, the microRNAs (miRNAs) are small noncoding RNAs of about 22 nt that modulate gene expression to repress target mRNAs to affect oncogenes or tumor inhibitor genes [9]. As one of the miRNAs, microRNA-143 (miR-143) has been identified as a tumor repressor [10], and it has been demonstrated that miR-143 was implicated in the progression of BC [11–13]. Besides, it has been demonstrated that resistance to ralstonia solanacearum 1 (RRS1) serves as a ribosome biogenesis protein in yeast and plants [14], which has also been clarified to be associated with BC [15, 16].

The lncRNAs were recently defined as competing endogenous RNAs (ceRNAs) of miRNAs to affect the tumor progression, and lncRNA SBF2-AS1 has been suggested to participate in the development of human cancers, such as liver cancer, cervical cancer, esophageal squamous carcinoma, non-small cell lung cancer and gastric cancer [8, 17–20]. However, the functional mechanism of lncRNA SBF2-AS1 in BC remains unknown. In order to fill the research gap, here we aim to investigate the role of lncRNA SBF2-AS1 as a sponge absorbing miR-143 in BC progression by regulating RRS1. We studied whether lncRNA SBF2-AS1 could be a novel target for BC treatment, thereby helping to find efficient therapeutic strategies for BC, and we deduced that SBF2-AS1 could serve as a ceRNA to modulate the tumorigenesis and progression of BC by regulating miR-143 and RRS1.

## Materials and methods

### Ethics statement

Written informed consents were acquired from all patients prior to the study. The protocols of this study were affirmed by the Ethic Committee of Tongji Hospital, Tongji Medical College, Huazhong University of Science and Technology and based on the ethical principles for medical research involving human subjects of the Helsinki Declaration. Animal experiments were strictly in line with the Guide to the Management and Use of Laboratory Animals issued by the National Institutes of Health. The protocol of animal experiments was approved by the Institutional Animal Care and Use Committee of Tongji Hospital, Tongji Medical College, Huazhong University of Science and Technology.

### Study subjects

A total of 50 BC tissues from BC patients (mean age of 52.50 ± 6.89 years old) that have received operative treatment in Tongji Hospital, Tongji Medical College, Huazhong University of Science and Technology from January 2016 to January 2017 were collected, and 50 adjacent normal tissues were also harvested (over 5 cm from the cancer tissue). The pathological patterns of the BC samples were all invasive BC. Patients that have received neoadjuvant chemotherapy, radiotherapy or endocrinotherapy were excluded. The general information, pathological diagnosis and treatment information of the BC patients were analyzed.

### Cell culture

Normal mammary epithelial cell line MCF-10A and BC cell lines (MCF-7 and MDA-MB-231) were all obtained from Shanghai Institutes for Biological Sciences, Chinese Academy of Sciences (Shanghai, China), and were cultured in Dulbecco’s modified Eagle medium (DMEM) containing 10% fetal bovine serum (FBS) in an incubator with 95% air and 5% CO_2_, and the temperature was set at 37 °C. After the cells were detached by 0.25% trypsin and passaged, the expression of SBF2-AS1 and miR-143 in cells were evaluated using reverse transcription quantitative polymerase chain reaction (RT-qPCR), and the mRNA and protein expression of RRS1 were assessed by RT-qPCR and Western blot analysis.

### Cell grouping and transfection

MDA-MB-231 and MCF-7 cells were selected to explore the impacts of SBF2-AS1 on BC cells, which were separated into 7 groups: the blank group (the cells without any transfection); the si-negative control (NC) group (cells were transfected with silenced SBF2-AS1 NC vector); the si-SBF2-AS1 group (cells were transfected with silenced SBF2-AS1 vector); the mimics NC group (cells were introduced with miR-143 mimics NC); the miR-143 mimics group (cells were introduced with miR-143 mimics); the overexpressed (oe)-SBF2-AS1 + mimics NC group (cells were transfected with oe-SBF2-AS1 vector and miR-143 mimics NC); the oe-SBF2-AS1 + miR-143 mimics group (cells were transfected with oe-SBF2-AS1 vector and miR-143 mimics). Mimics NC, miR-143 mimics, si-NC, si-SBF2-AS1 and oe-SBF2-AS1 were all designed and synthesized by Shanghai GenePharma Co., Ltd. (Shanghai, China). The medium was changed to the serum-free medium that without penicillin and streptomycin in the transfection. According to the instructions of Lipofectamine™ 2000 reagent, the silcenced or overexpressed plasmids, and mimics or its NC were mixed and placed for 20 min. The medium was replaced by normal complete medium after the cells were transfected for 6–8 h.

### 5-Ethynyl-2′-deoxyuridine (EdU) assay

Cells in the logarithmic growth phase were collected after transfection, and were seeded onto 96-well plates at 6000 cells/well for 24-h incubation. When the cell confluence reached 60%, each well was incubated with 100 μL diluted EdU solution for 2 h. The EdU kits were purchased from Guangzhou RiboBio Co., Ltd. (Guangdong, China). The cells were fixed and stained based on the instructions of the EdU kits. After observed and photographed under a fluorescence microscope, cells in four random fields of view were counted, and the proliferation rate was calculated.

### Colony formation assay

Cells in the logarithmic growth phase were detached by trypsin and made into cell suspension after transfection. The counted cells were paved on the 6-well plates at 300 cells/well. Three duplicate wells were set in each group. After cultured for 14 d, the cells were washed by phosphate buffered saline (PBS) 3 times. Next, the cells were successively fixed by 4% paraformaldehyde and stained with 0.1% crystal violet staining solution for 30 min. With the dye removed, the colony formation rate was calculated under an inverted microscope.

### 3-(4,5-dimethyl-2-thiazolyl)-2,5-diphenyl-2-H-tetrazolium bromide (MTT) assay

Cells in the logarithmic growth phase were detached by trypsin and made into cell suspension after transfection. Afterwards, the cells were seeded onto 96-well plates at 1 × 10^5^ cells/well, each well was appended with 200 μL medium and marked, and the cells were incubated with 5% CO_2_ at 37 °C for 48 h. After the medium was removed, each well was supplemented with 100 μL serum-free DMEM and 10 μL MTT solution, and the plates were incubated with 5% CO_2_ at 37 °C for 4 h. Next, every well was added with 100 μL dimethyl sulfoxide, and the absorbance (A) value at 490 nm of each well was determined, the larger A value expressed for higher cell viability.

### Flow cytometry

Cells in the logarithmic growth phase were detached by trypsin and made into cell suspension after transfection. Then the cells were resuspended by 70% ethanol, fixed at 4 °C overnight, and centrifuged at 1000 r/min. Subsequently, the cells were appended with 100 μL RNAse, incubated in water bath at 37 °C for 30 min, and supplemented with 100 μL propidium iodide (PI), then incubated at 4 °C without light exposure for 30 min. The cell cycle was analyzed by flow cytometer.

Cells in the logarithmic growth phase were collected after transfection, and detached by trypsin, then made into cell suspension. Next, the cells were fixed by cold ethanol at 4 °C overnight, centrifuged for 5 min, and added with 5 μL Annexin V-fluoresceine isothiocyanate. After 3 min, the cells were then appended with 10 μL PI, and incubated at 37 °C without light exposure for 15 min. Afterwards, the cells were centrifuged and resuspended in 0.5 mL pre-cooled buffer solution, and the cell apoptosis was observed by flow cytometer.

### Transwell assay

Cells in the logarithmic growth phase were collected after transfection, and treated with serum-free medium for more than 8–12 h. The Transwell chambers were coated with matrigel (matrigel was used in invasion experiment but not in the migration experiment). The cells were detached by trypsin, and 5 × 10^4^ cells were suspended by 250 μL serum-free medium, incubated at 37 °C for 48 h. Next, the cells were fixed by 4% paraformaldehyde for 15 min, stained by 0.1% crystal violet staining solution for 15 min, and photographed under a microscope.

### Subcutaneous tumorigenesis in nude mice

Eighty-four BALB/c nude mice (aging 4–5 w, weighing 15–30 g) were all acquired from Shanghai Laboratory Animal Center, Chinese Academy of Sciences (Shanghai, China). The nude mice were fed in specific pathogen-free environment (temperature at 18–23 °C, humidity at 50–60%, and 12 h day/night cycle) for 1 w, the food and water were all disinfected. The nude mice were randomly divided into 14 groups (6 nude mice in each group): the blank group (the nude mice were injected with MDA-MB-231 or MCF-7 cells without any transfection); the si-NC group (the nude mice were injected with MDA-MB-231 or MCF-7 cells with silenced SBF2-AS1 NC vector); the si-SBF2-AS1 group (the nude mice were injected with MDA-MB-231 or MCF-7 cells with silenced SBF2-AS1 vector); the mimics NC group (the nude mice were injected with MDA-MB-231 or MCF-7 cells with miR-143 mimics NC); the miR-143 mimics group (the nude mice were injected with MDA-MB-231 or MCF-7 with miR-143 mimics); the oe-SBF2-AS1 + mimics NC group (the nude mice were injected with MDA-MB-231 or MCF-7 cells with oe-SBF2-AS1 vector and miR-143 mimics NC); the oe-SBF2-AS1 + miR-143 mimics group (the nude mice were injected with MDA-MB-231 or MCF-7 cells with oe-SBF2-AS1 vector and miR-143 mimics). The cells were made into cell suspension by trypsin, and the cell density was adjusted into 1 × 10^7^ cells/mL. The nude mice were partially disinfected and subcutaneously injected with 0.5 mL cell suspension at the root of thigh, then general circumstance of the nude mice was observed, and the tumors were measured by a vernier caliper every 5 d. After injected for 25 d, the nude mice were euthanized with their tumors extracted, and the weight of the tumors was measured, based on which the growth curve was graphed, and the tumor weight of each group was compared.

### Immunohistochemical staining

The xenografts were cut into 3 μm thick sections, and the sections were normally embedded by paraffin, toasted, dewaxed and hydrated according to the instructions. After toasted at 600 °C in a drying oven (DHG-9140A, Shanghai Huitai Instrument Manufacturing Co., Ltd., Shanghai, China) for 12 h, the sections were conducted with Ventana BenchMark GX staining (Ventana Medical Systems, Inc., AZ, USA) to evaluate the expression of Ki-67. The Ki-67 rabbit anti-human monoclonal antibody (GB13030–2), secondary antibody (GB23204) and diaminobenzidine (DAB) buffer solution (G1211) were all acquired from Servicebio Co., Ltd. (Hubei, China), and the steps were in line with the kit directions. The nuclei were stained into blue by hematoxylin and the DAB positive expression was brown or brownish-yellow. The results were evaluated by semi quantitative integration method and the brown or brownish-yellow particles were defined as positive cells; (1) staining intensity as the standard: unstained, 0 score; pale yellow, 1 score; brownish-yellow, 2 scores; brown, 3 scores; (2) percentage of stained cells in total cells as the standard: ≤ 5%, 0 score; 6–25%, 1 score; 26–50%, 2 scores; 51–75%, 3 scores; ≥ 76%, 4 scores. The score of each sample was calculated as the product of scores in (1) and (2).

### Terminal deoxynucleotidyl transferase-mediated dUTP nick end-labeling (TUNEL) staining

The paraffin sections were dewaxed and added with proteinase K at 30 °C for 20 min, then incubated with endogenous peroxidase blocking buffer for 5 min. After stained with TUNEL solution (Beyotime Institute of Biotechnology, Shanghai, China) at 37 °C without light exposure for 60 min, the sections were developed by DAB solution and counterstained by hematoxylin. The TUNEL positive cells were brown while the normal cells were blue.

### Fluorescence in situ hybridization (FISH)

The subcellular localization of SBF2-AS1 was assessed by FISH technique according to the direction of Ribo™ lncRNA FISH Probe Mix (Red) (Guangzhou RiboBio Co., Ltd., Guangdong, China). The cells were seeded onto 24-well plates at 6 × 10^4^ cells/well, when the cell confluence reached 80%, cells were fixed by 1 mL 4% paraformaldehyde, treated with proteinase K, glycine, and acetylation reagent, then appended with 250 μL pre-hybridization solution and incubated at 42 °C for 1 h. With the pre-hybridization solution removed, the cells were supplemented with 250 μL SBF2-AS1 hybridization solution containing probe (300 ng/mL) at 42 °C overnight. Afterwards, the cells were stained by phosphate buffered solution with tween (PBST)-diluted 4′,6-diamidino-2-phenylindole 2 hci (ab104139, 1: 100, Abcam Co., Ltd., Shanghai, China) for 5 min on 24-well plates. After washed by PBST for 3 times (3 min/time), the cells were sealed by anti-fluorescence quencher, then observed and photographed by a fluorescence microscope (Olympus Optical Co., Ltd., Tokyo, Japan).

### RT-qPCR

The total RNA in tissues and cells were extracted by Trizol kits (Invitrogen, Carlsbad, CA, USA), and the concentration and optical density (OD) were assessed by a spectrophotometer. The value of RNA_A260nm/A280nm_ that ranged from 1.8 to 2.0 indicated a well purity of the extracted RNA. Next, the RNA of mRNA and lncRNA was reversely transcripted into cDNA by GoldScript one-step RT-PCR Kit (Applied Biosystems, Carlsbad, CA, USA), and the RNA of miRNA was reversely transcripted into cDNA by Hairpin-itTM miRNA detection kits (Shanghai GenePharma Co., Ltd., Shanghai, China). The PCR was conducted by SYBR premix Ex Taq™ II PCR Kit (TaKaRa Biotechnology Co. Ltd., Liaoning China) on the ABI7500 PCR instrument. The primers (Table 1) were designed and synthesized by Beijing ComWin Biotech Co., Ltd. (Beijing, China), U6 and glyceraldehyde phosphate dehydrogenase (GAPDH) were taken as the internal references. The data were analyzed by 2^-△△Ct^ method.

**Table 1:** Primer sequence

Gene	Primer sequence (5′ → 3′)
MiR-143	F: TGAGATGAAGCACTGTAGCTC
	R: GCGAGCACAGAATTAATACGAC
U6	F: TTATGGGTCCTAGCCTGAC
	R: CACTATTGCGGGCTGC
SBF2-AS1	F: AGACCATGTGGACCTGTCACTG
	R: GTTTGGAGTGGTAGAAATCTGTC
RRS1	F: CCGAAAAGGGGTTGAAACTTCC
	R: CCCTACCGGACACCAGAGTAA
GAPDH	F: CCACATCGCTCAGACACCAT
	R: ACCAGGCGCCCAATACG

*F* forward, *R* reverse, *miR-143* microRNA-143, *SBF2-AS1* SET-binding factor 2-antisense RNA1, *RRS1* resistance to ralstonia solanacearum 1, *GAPDH* glyceraldehyde phosphate dehydrogenase

### Western blot analysis

The total protein in tissues and cells was extracted, which was then added into 1/4 volume of 5 × sodium dodecyl sulfate buffer solution at 100 °C for 5 min, conducted with electrophoresis by 12% separation gel and 4% spacer gel, and transferred onto the membranes. Consequently, the membranes were blocked by bovine serum albumin that had been diluted by tris buffer solution with tween for 60 min. The membranes were added with primary antibodies RRS1 (1: 1000), Bax (1:1000), Bcl-2 (1: 2000), Ki-67 (1: 5000), CyclinD1 (1: 1000), matrix metalloprotease (MMP)-2 (1: 500) and MMP-9 (1: 1000) (all from Abcam, Cambridge, MA, USA) at 4 °C overnight after the transfection. Next, the membranes were incubated with relative secondary antibodies for 2 h. After developed by enhanced chemiluminescent and exposure, the gray values of the protein bands were analyzed by software.

### Dual luciferase reporter gene assay

The binding sites between SBF2-AS1 and miR-143 were predicted by a bioinformatic website (https://cm.jefferson.edu/rna22/Precomputed/), and the binding relation between SBF2-AS1 and miR-143 was evaluated by dual luciferase reporter gene assay. The gene fragment of synthesized SBF2-AS1 3′-untranslated region (3’UTR) was introduced into pMIR-reporter (Huayueyang Biotechnology Co., Ltd., Beijing, China) by endonuclease sites Bamh1 and Ecor1. Mutation sites of complementary sequence of the seed sequence was designed on SBF2-AS1 wild type (WT), which were then digested by restriction endonuclease, and the target fragment was inserted into pMIR-reporter plasmid by T4 DNA ligase. The correctly identified luciferase reporter plasmids WT and mutation type (MUT) with mimics NC and miR-143 mimics were co-transfected into MDA-MB-231 and MCF-7 cells. After 48-h transfection, the cells were lysed, and the luciferase activity was assessed by luciferase detection kits (BioVision, San Francisco, CA, USA) and Glomax20/20 luminometer (Promega, Madison, WI, USA).

The target relation between miR-143 and RRS1, as well as the binding sites between miR-143 and RRS1 3’UTR were predicted by a bioinformatic software (http://www.targetscan.org). RRS1 3’UTR promoter region sequence containing binding sites of miR-143 was synthesized, and RRS1-WT was established, based on which the binding sites were mutated, thereby RRS1-MUT was established. MDA-MB-231 and MCF-7 cells in the logarithmic growth phase were seeded onto 96-well plates, when the cell confluence reached 70%, RRS1-WT and RRS1-MUT with mimics NC and miR-143 mimics were co-transfected into MDA-MB-231 and MCF-7 cells. After 48-h transfection, the cells were lysed, and the luciferase activity was measured by luciferase detection kits.

### RNA pull-down assay

The cells were respectively transfected with biotin-labeled miR-143 WT plasmid (50 nM) and biotin-labeled miR-143 MUT plasmid (50 nM) for 48 h, and cultured by lysis solution (Ambion, Company, Austin, TX, USA) for 10 min, then 50 mL cell lysis was subpackaged. The remained lysate was co-cultured with M-280 streptavidin magnetic beads that have been pre-coated by RNase-free and yeast tRNA (all from Sigma, St. Louis, MO, USA) at 4 °C for 3 h. Antagonism miR-143 probe was taken as the NC, the total RNA was extracted by Trizol, and the expression of SBF2-AS1 was evaluated by RT-qPCR.

### Statistical analysis

All data analyses were conducted using SPSS 21.0 software (IBM Corp. Armonk, NY, USA). The enumeration data were expressed as rate or percentage, and analyzed by chi-square test or Fisher exact test. The measurement data conforming to the normal distribution were expressed as mean ± standard deviation. The t-test was performed for comparisons between two groups and one-way analysis of variance (ANOVA) was used for comparisons among multiple groups, and the Fisher’s least significant difference t (LSD-t) test was used for pairwise comparisons after one-way ANOVA. *P* value < 0.05 was indicative of statistically significant difference.

## Results

### General data analysis of study subjects

We have found in the general data analysis of study subjects (Table 2) that the mean age of patients in the BC group was 52.50 ± 6.89 years, the main histological type was ductal carcinoma (90%), and the major histological grade was stage II (52%), followed by stage I (28%) and stage III (20%); as for LNM, patients with LNM accounted for 60%, and patients without LNM made up for 40%; the tumor, node and metastasis (TNM) stage successively was stage II (72%), stage III (20%) and stage I (8%); the tumor size of 62% patients was ≥2 cm, and that of 38% patients was < 2 cm; the clinical stage successively was stage II (56%), stage III (34%) and stage I (10%).

**Table 2:** General data analysis of BC patients

Characteristic	Value
Age (year)	52.50 ± 6.89
Histological type	
Ductal	90% (45)
Lobular	4% (2)
Other	6% (3)
Histological grade	
I	28% (14)
II	52% (26)
III	20% (10)
Lymph node metastasis	
Yes	40% (20)
No	60% (30)
TNM stage	
I	8% (4)
II	72% (36)
III	20% (10)
Tumor size	
< 2 cm	38% (19)
≥ 2 cm	62% (31)
Clinical stage	
I	10% (5)
II	56% (28)
III	34% (17)

### SBF2-AS1 and RRS1 are highly expressed, and miR-143 is poorly expressed in BC tissues

**Fig. 1:**
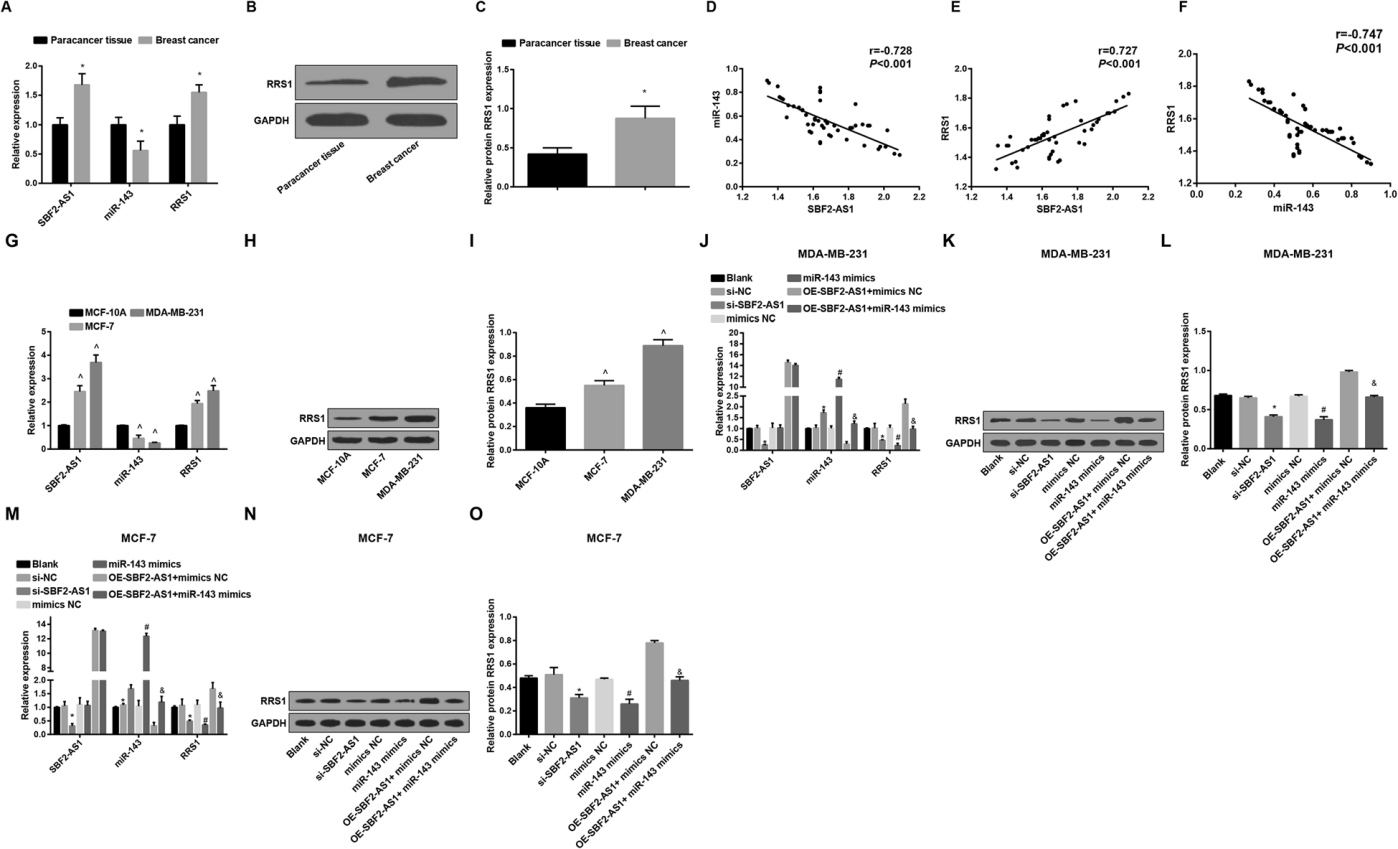
SBF2-AS1 and RRS1 are highly expressed, and miR-143 is poorly expressed in BC tissues and cell lines. **a** The expression of SBF2-AS1, miR-143 and RRS1 in BC tissues and adjacent normal tissues; **b** Protein band of RRS1 in BC tissues and adjacent normal tissues; **c** Statistical results of protein expression of RRS1; **d** The relation between SBF2-AS1 expression and miR-143 expression in BC patients was analyzed by Pearson correlation analysis; **e** The relation between SBF2-AS1 expression and RRS1 expression in BC patients was analyzed by Pearson correlation analysis; **f** Relation between miR-143 expression and RRS1 expression in BC patients was analyzed by Pearson correlation analysis. **g** The expression of SBF2-AS1, miR-143 and RRS1 in MCF-10A, MCF-7 and MDA-MB-231 cells; **h** Protein bands of RRS1 in MCF-10A, MCF-7 and MDA-MB-231 cells in Western blot analysis; **i** Statistical results of the protein expression of RRS1; **j** Expression of SBF2-AS1, miR-143 and RRS1 in MDA-MB-231 cells; **k** Protein band of RRS1 in MDA-MB-231 cells; **l** Protein expression of RRS1 in MDA-MB-231 cells; **m** Expression of SBF2-AS1, miR-143 and RRS1 in MCF-7 cells; **n** Protein band of RRS1 in MCF-7 cells; **o** Protein expression of RRS1 in MCF-7 cells. The measurement data conforming to the normal distribution were expressed as mean ± standard deviation. **a–c**: The t-test was performed for comparisons between two groups, *n* = 50, * *P* < 0.05 vs the adjacent normal tissues. **g–o**: One-way ANOVA was used for comparisons among multiple groups, and LSD-t test was used for pairwise comparisons after one-way ANOVA, *N* = 3, ^ *P* < 0.05 vs the MCF-10A cells; * *P* < 0.05 vs the si-NC group; # *P* < 0.05 vs the mimics NC group; & *P* < 0.05 vs the oe-SBF2-AS1 + mimics NC group

The expression of SBF2-AS1, RRS1 and miR-143 in BC tissues and adjacent normal tissues was assessed by RT-qPCR, the results (Fig. 1a) reflected that relative to the adjacent normal tissues, the mRNA expression of SBF2-AS1 and RRS1 was elevated, and miR-143 expression was repressed in the BC tissues (all *P* < 0.05). Outcomes of Western blot analysis (Fig. 1b–c) revealed that the protein expression of RRS1 was enhanced in the BC tissues (*P* < 0.05).

Pearson correlation analysis was employed to analyze the relation among the expression of SBF2-AS1, miR-143 and RRS1, the results (Fig. 1d–f) implied that SBF2-AS1 expression was negatively related with miR-143 expression (*r* = − 0.728), SBF2-AS1 expression was in positive correlation with the mRNA expression of RRS1 (*r* = 0.727), and miR-143 was negatively correlated with the mRNA expression of RRS1 (*r* = − 0.747, all *P* < 0.001).

### SBF2-AS1 expression is related with LNM, tumor size and clinical stage of the BC patients

The relation between SBF2-AS1 expression and clinicopathological features of BC patients was analyzed, the results (Table 3) unveiled that: the mean value of relative expression of SBF2-AS1 in BC tissues was taken as the demarcation line, the 50 BC tissues were divided into the SBF2-AS1 high expression group (*n* = 39) and the SBF2-AS1 low expression group (*n* = 11). The correlation between SBF2-AS1 and different clinicopathological parameters of BC patients was analyzed by chi-square test or Fisher exact test. The outcomes reflected that SBF2-AS1 expression was not related with age, histological type, histological grade and TNM stage (all *P* > 0.05), while was correlated with LNM, tumor size and clinical stage of BC patients (all *P* < 0.05).

**Table 3:** Relation between clinicopathological parameter and SBF2-AS1 expression of BC patients

Clinicopathological parameter		SBF2-AS1		*P* value
		High expression (*n* = 39)	Low expression (*n* = 11)	
Age (years)				
< 50	29	24	5	0.491
≥ 50	21	15	6	
Histological type				
Ductal	45	36	9	0.167
Lobular	2	1	2	
Other	3	2	1	
Histological grade				
I	14	10	4	0.142
II	26	23	3	
III	10	6	4	
Lymph node metastasis				
YES	20	12	8	0.012
NO	30	27	3	
TNM stage				
I	4	3	1	0.977
II	36	28	8	
III	10	8	2	
Tumor size				
< 2 cm	19	10	9	0.001
≥ 2 cm	31	29	2	
Clinical stage				
I	5	1	4	0.004
II	28	24	4	
III	17	14	3	

### SBF2-AS1 and RRS1 are highly expressed, and miR-143 is poorly expressed in BC cell lines

The expression of SBF2-AS1 and miR-143 in normal mammary epithelial cell line MCF-10A and BC cell lines (MCF-7 and MDA-MB-231) was measured by RT-qPCR, we have found that (Fig. 1g) SBF2-AS1 expression in BC cell lines was broadly higher and miR-143 expression was markedly lower than in the normal mammary epithelial cell line MCF-10A, showing that SBF2-AS1 was upregulated, and miR-143 was poorly expressed in BC cells (all *P* < 0.05).

RRS1 expression in normal mammary epithelial cell line MCF-10A and BC cell lines (MCF-7 and MDA-MB-231) was assessed by RT-qPCR and Western blot analysis, the results (Fig. 1h–i) suggested that RRS1 expression in BC cell lines MCF-7 and MDA-MB-231 was amplified, which was relative to that in normal mammary epithelial cell line MCF-10A, indicating that RRS1 was highly expressed in BC cells (*P* < 0.05).

The expression levels of SBF2-AS1, miR-143 and RRS1 in MDA-MB-231 and MCF-7 cells were determined by RT-qPCR and Western blot analysis, we have found that (Fig. 1j–o) there were no significant difference in the expression of SBF2-AS1 and miR-143 among the blank group, the si-NC group and the mimics NC group, and also in RRS1 expression among the blank group, the si-NC group, the mimics NC group and the oe-SBF2-AS1 + miR-143 mimics group (all *P* > 0.05); in contrast to the si-NC group, levels of SBF2-AS1 and RRS1 were declined, while miR-143 expression was augmented in the si-SBF2-AS1 group; relative to the mimics NC group, miR-143 expression was enhanced while RRS1 expression was decreased in the miR-143 mimics group; in comparison to the oe-SBF2-AS1 + mimics NC group, miR-143 expression was heightened while RRS1 expression was suppressed in the oe-SBF2-AS1 + miR-143 mimics group (all *P* < 0.05).

### SBF2-AS1 competitively regulates miR-143 and RRS1 is targeted by miR-143

**Fig. 2:**
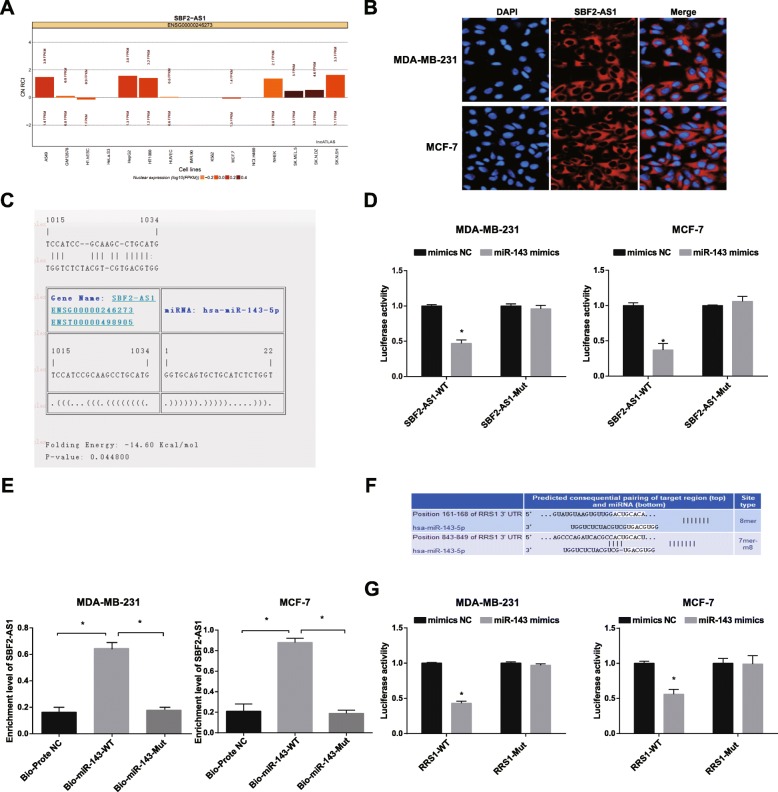
SBF2-AS1 competitively regulates miR-143 and RRS1 is targeted by miR-143. **a** the Subcellular localization of SBF2-AS1 was analyzed by online prediction website; **b** the subcellular localization of SBF2-AS1 was confirmed by FISH assay; **c** The binding sites between SBF2-AS1 and miR-143 were predicted by RNA22 website; **d** The binding relation between SBF2-AS1 and miR-143 was affirmed by dual luciferase reporter gene assay; **e** The enrichment of miR-143 on SBF2-AS1 was detected by RNA pull-down assay; **f** The binding sites of RRS1 and miR-143 were predicted by http://www.targetscan.org; **g** The binding relation between miR-143 and RRS1 was affirmed by dual luciferase reporter gene assay. * *P* < 0.05, The measurement data conforming to the normal distribution were expressed as mean ± standard deviation. The t-test was performed for comparisons between two groups, *N* = 3

To investigate the function mechanism of SBF2-AS1, we firstly employed online analysis website (http://lncatlas.crg.eu/), the results (Fig. 2a) revealed that SBF2-AS1 was mainly expressed in cytoplasm, which was confirmed again by RNA-FISH assay (Fig. 2b), reflecting that SBF2-AS1 functioned in cytoplasm. The outcomes that analyzed by RNA22 website (https://cm.jefferson.edu/rna22/Precomputed/) revealed that SBF2-AS1 could bind to miR-143 (Fig. 2c–d). According to the results of dual luciferase reporter gene assay, the luciferase activity was degraded in the WT SBF2-AS1 + miR-143 mimics group, which was relative to the mimics NC group (*P* < 0.05), while the luciferase activity didn’t obviously change in the MUT SBF2-AS1 + miR-143 mimics group (*P* > 0.05), suggesting that there existed binding relation between SBF2-AS1 and miR-143. The competitive adsorption of SBF2-AS1 on miR-143 was affirmed by RNA pull-down assay, and the outcomes (Fig. 2e) unraveled that relative to the Bio-probe NC group, the enrichment of SBF2-AS1 was elevated in the Bio-miR-143-WT group (*P* < 0.05), while no observable difference could be found in the enrichment of SBF2-AS1 between the Bio-miR-143-MUT group and the Bio-probe NC group (*P* > 0.05). The above results indicated that SBF2-AS1 could adsorb miR-143 as a ceRNA, thereby modulate the expression of miR-143.

The binding sites between RRS1 and miR-143 were predicted by online prediction software (http://www.targetscan.org), the binding sequence of 3′-UTR of RRS1 mRNA and miR-143 was shown in Fig. 2f. The outcomes of dual luciferase reporter gene assay (Fig. 2g) indicated that miR-143 mimics exerted no evident impact on the luciferase activity of MUT-miR-143/RRS1 plasmid (*P* > 0.05), while the luciferase activity of WT-miR-143/RRS1 plasmid was reduced (*P* < 0.05).

### Reduced SBF2-AS1 and overexpressed miR-143 inhibit the viability of BC cells

**Fig. 3:**
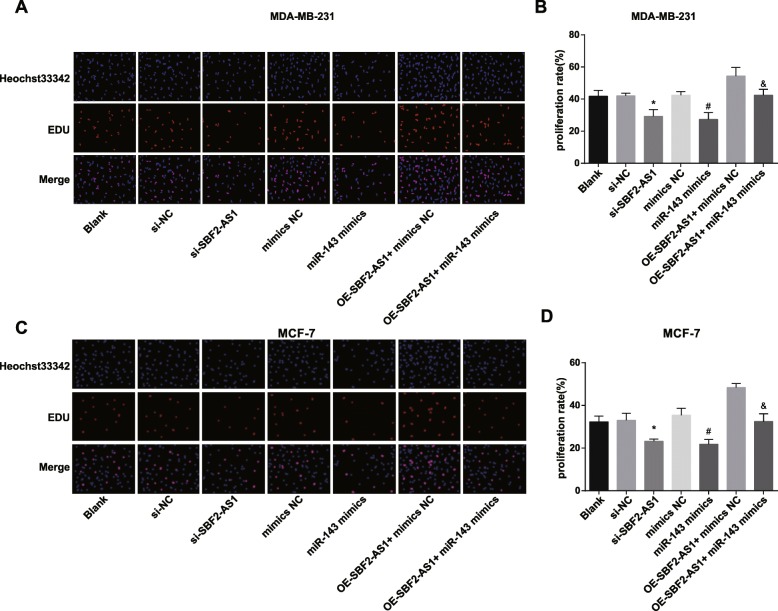
Reduced SBF2-AS1 and overexpressed miR-143 prohibit the proliferation of BC cells. **a** Proliferation of MDA-MB-231 cells was detected by EdU assay; **b** Statistical results of MDA-MB-231 cell proliferation of each group; **c** Proliferation of MCF-7 cells was detected by EdU assay; **d** Statistical results of MCF-7 cell proliferation of each group; The measurement data conforming to the normal distribution were expressed as mean ± standard deviation. One-way ANOVA was used for comparisons among multiple groups, and LSD-t test was used for pairwise comparisons after one-way ANOVA, *N* = 3, * *P* < 0.05 vs the si-NC group; # *P* < 0.05 vs the mimics NC group; & *P* < 0.05 vs the oe-SBF2-AS1 + mimics NC group

**Fig. 4:**
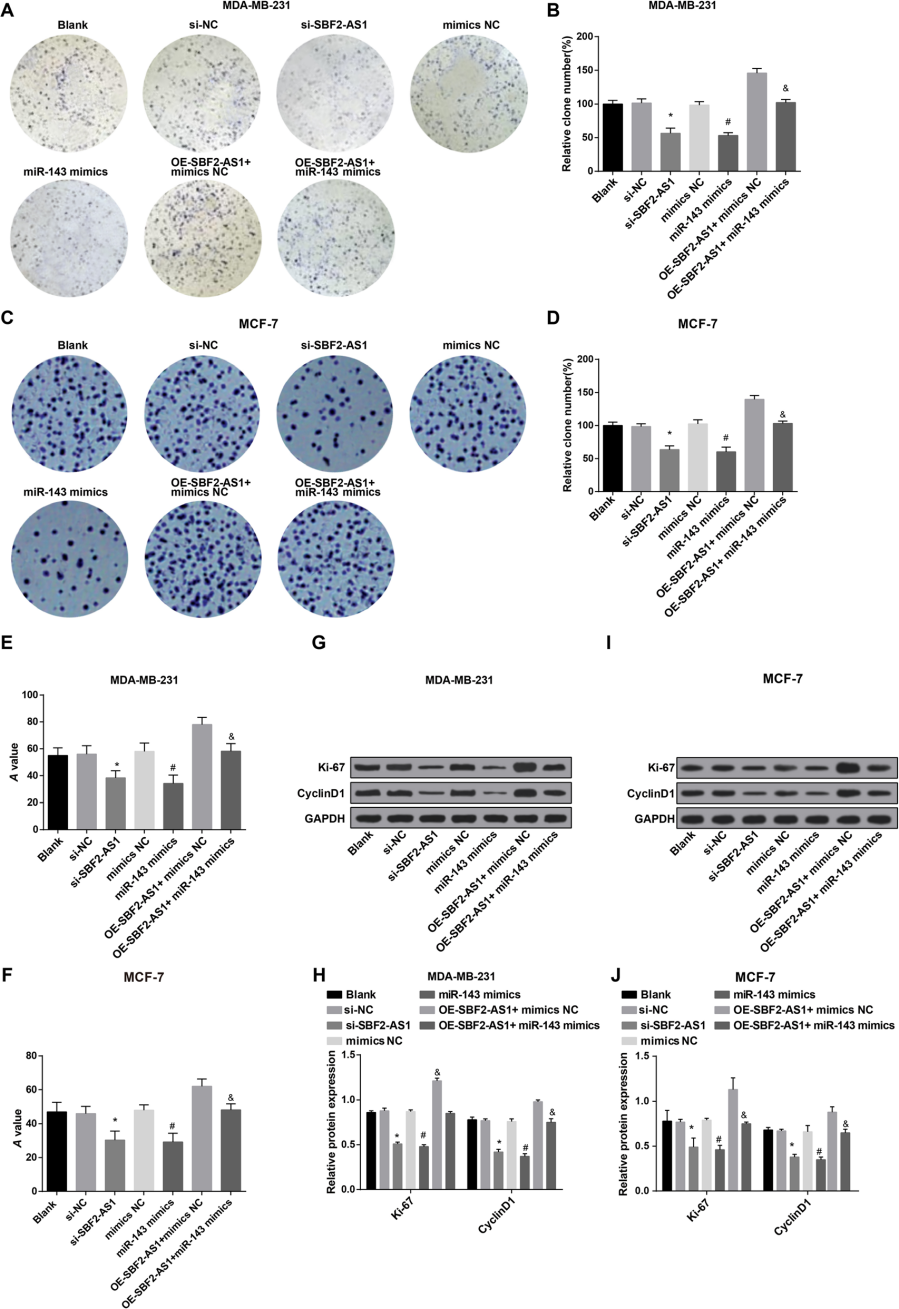
Reduced SBF2-AS1 and overexpressed miR-143 prohibit the viability of BC cells. **a** The colony formation ability of MDA-MB-231 cells in each group was detected by colony formation assay; **b** Statistical results of colony formation ability of MDA-MB-231 cells in each group; **c** The colony formation ability of MCF-7 cells in each group was detected by colony formation assay; **d** Statistical results of colony formation ability of MCF-7 cells in each group; **e** MDA-MB-231 cell viability was detected by MTT assay; **f** MCF-7 cell viability was detected by MTT assay; **g** Protein bands of Ki-67 and CyclinD1 in MDA-MB-231 cells in Western blot analysis; **h** Statistical results of the protein expression of Ki-67 and CyclinD1 in MDA-MB-231 cells; **i** Protein bands of Ki-67 and CyclinD1 in MCF-7 cells in Western blot analysis; **j** Statistical results of the protein expression of Ki-67 and CyclinD1 in MCF-7 cells. The measurement data conforming to the normal distribution were expressed as mean ± standard deviation. One-way ANOVA was used for comparisons among multiple groups, and LSD-t test was used for pairwise comparisons after one-way ANOVA, *N* = 3, * *P* < 0.05 vs the si-NC group; # *P* < 0.05 vs the mimics NC group; & *P* < 0.05 vs the oe-SBF2-AS1 + mimics NC group

EdU assay, colony formation assay and MTT assay were employed to measure the proliferation of MDA-MB-231 and MCF-7 cells, the results reflected that (Figs. 3,4a–f) there was no apparent difference in proliferation rate, colony formation rate and cell viability among the blank group, the si-NC group, the mimics NC group and the oe-SBF2-AS1 + miR-143 mimics group (all *P* > 0.05); in contrast to the si-NC group and the mimics NC group, the proliferation rate, colony formation rate and cell viability were reduced in the si-SBF2-AS1 group and the miR-143 mimics group; relative to the oe-SBF2-AS1 + mimics NC group, the proliferation rate, colony formation rate and cell viability were restrained in the oe-SBF2-AS1 + miR-143 mimics group (all *P* < 0.05).

The protein expression of Ki-67 and CyclinD1 in MDA-MB-231 and MCF-7 cells of each group was measured by Western blot analysis, the results unearthed that (Fig. 4g–j) no noticeable difference could be observed in protein expression of Ki-67 and CyclinD1 among the blank group, the si-NC group, the mimics NC group and the oe-SBF2-AS1 + miR-143 mimics group (all *P* > 0.05); contrasted to the si-NC group and the mimics NC group, the protein expression of Ki-67 and CyclinD1 was attenuated in the si-SBF2-AS1 group and the miR-143 mimics group; in contrast to the oe-SBF2-AS1 + mimics NC group, the protein expression of Ki-67 and CyclinD1 was declined in the oe-SBF2-AS1 + miR-143 mimics group (all *P* < 0.05).

### Reduced SBF2-AS1 and overexpressed miR-143 accelerate cell cycle arrest and apoptosis of BC cells

**Fig. 5:**
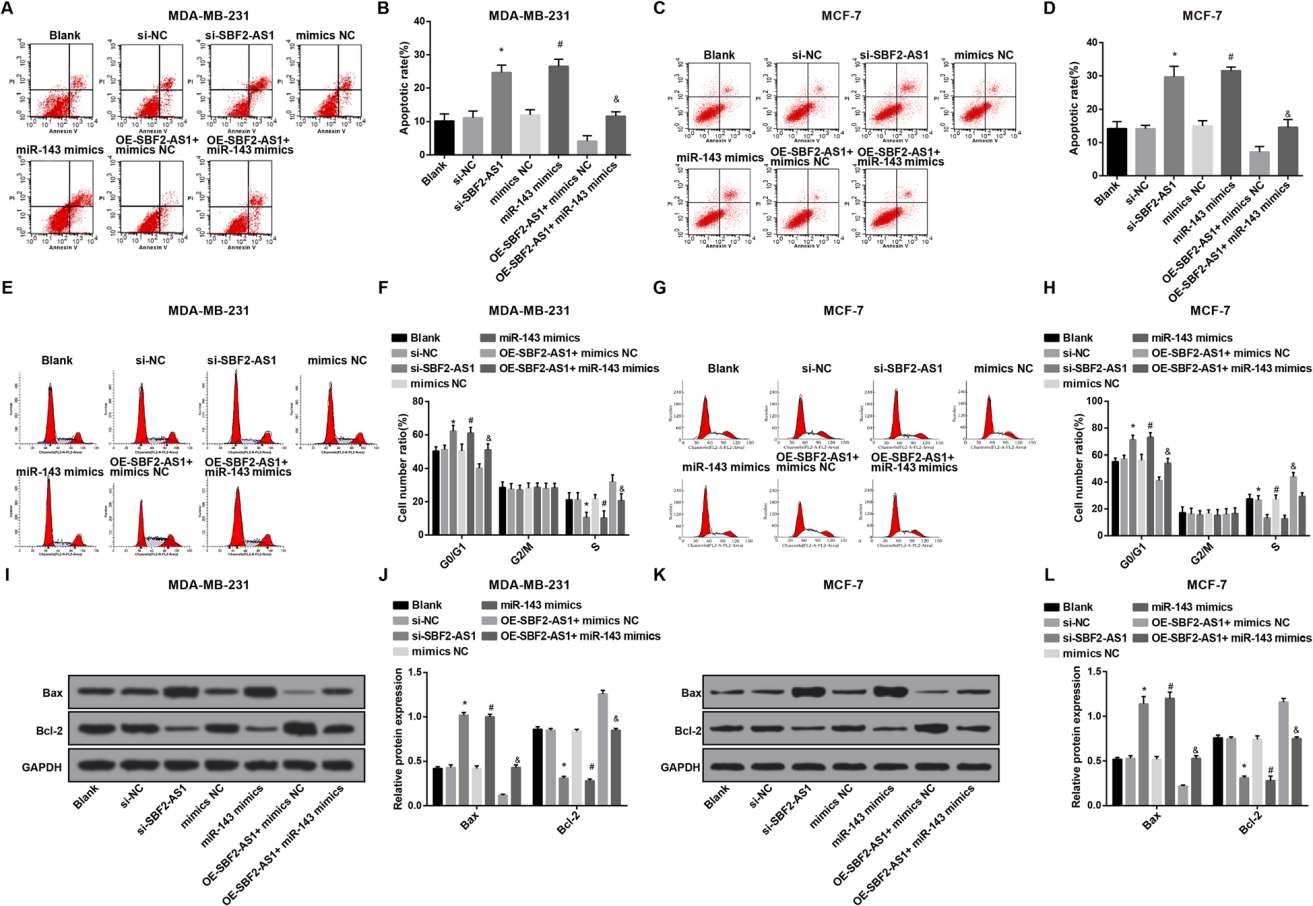
Reduced SBF2-AS1 and overexpressed miR-143 accelerate cell cycle arrest and apoptosis of BC cells. **a** Apoptotic rate of MDA-MB-231 cells in each group was detected by flow cytometry; **b** Statistical results of the apoptotic rate of MDA-MB-231 cells in each group; **c** Apoptotic rate of MCF-7 cells in each group was detected by flow cytometry; **d** Statistical results of the apoptotic rate of MCF-7 cells in each group; **e** Cell cycle distribution of MDA-MB-231 cells was detected by flow cytometry; **f** Statistical results of MDA-MB-231 cell cycle distribution of each group; **g** Cell cycle distribution of MCF-7 cells was detected by flow cytometry; **h** Statistical results of MCF-7 cell cycle distribution of each group; **i** Protein bands of Bax and Bcl-2 in MDA-MB-231 cells in Western blot analysis; **j** Statistical results of the protein expression of Bax and Bcl-2 in MDA-MB-231 cells; **k** Protein bands of Bax and Bcl-2 in MCF-7 cells in Western blot analysis; **l** Statistical results of the protein expression of Bax and Bcl-2 in MCF-7 cells. The measurement data conforming to the normal distribution were expressed as mean ± standard deviation. One-way ANOVA was used for comparisons among multiple groups, and LSD-t test was used for pairwise comparisons after one-way ANOVA, *N* = 3, * *P* < 0.05 vs the si-NC group; # *P* < 0.05 vs the mimics NC group; & *P* < 0.05 vs the oe-SBF2-AS1 + mimics NC group

The cell cycle arrest and apoptosis of MDA-MB-231 and MCF-7 cells were measured by flow cytometry, and we have discovered that (Fig. 5a–h) there was no remarkable difference could be found in cell cycle arrest and apoptosis of MDA-MB-231 and MCF-7 cells among the blank group, the si-NC group, the mimics NC group and the oe-SBF2-AS1 + miR-143 mimics group (all *P* > 0.05); relative to the si-NC group and the mimics NC group, MDA-MB-231 and MCF-7 cells in G0/G1 stage were elevated, cells in S stage was reduced, and the apoptotic rate was increased in the si-SBF2-AS1 group and the miR-143 mimics group, while cells in G2/M stage didn’t significantly change; in comparison to the oe-SBF2-AS1 + mimics NC group, MDA-MB-231 cells in G0/G1 stage were augmented, cells in S stage was reduced, and the apoptotic rate was boosted in the oe-SBF2-AS1 + miR-143 mimics group (all *P* < 0.05), while cells in G2/M stage didn’t significantly change.

The protein expression of Bax and Bcl-2 in MDA-MB-231 and MCF-7 cells were measured by Western blot analysis, the outcomes indicated that (Fig. 5i–l) there was no broad difference could be found in the protein expression of Bax and Bcl-2 in MDA-MB-231 and MCF-7 cells among the blank group, the si-NC group, the mimics NC group and the oe-SBF2-AS1 + miR-143 mimics group (all *P* > 0.05); relative to the si-NC group and the mimics NC group, the protein expression of Bcl-2 in MDA-MB-231 and MCF-7 cells was declined, and that of Bax was amplified in the si-SBF2-AS1 group and the miR-143 mimics group; contrasted to the oe-SBF2-AS1 + mimics NC group, protein expression of Bcl-2 in MDA-MB-231 and MCF-7 cells was suppressed, and that of Bax was enhanced in the oe-SBF2-AS1 + miR-143 mimics group (all *P* < 0.05).

### Reduced SBF2-AS1 and overexpressed miR-143 restrain the invasion and migration of BC cells

**Fig. 6:**
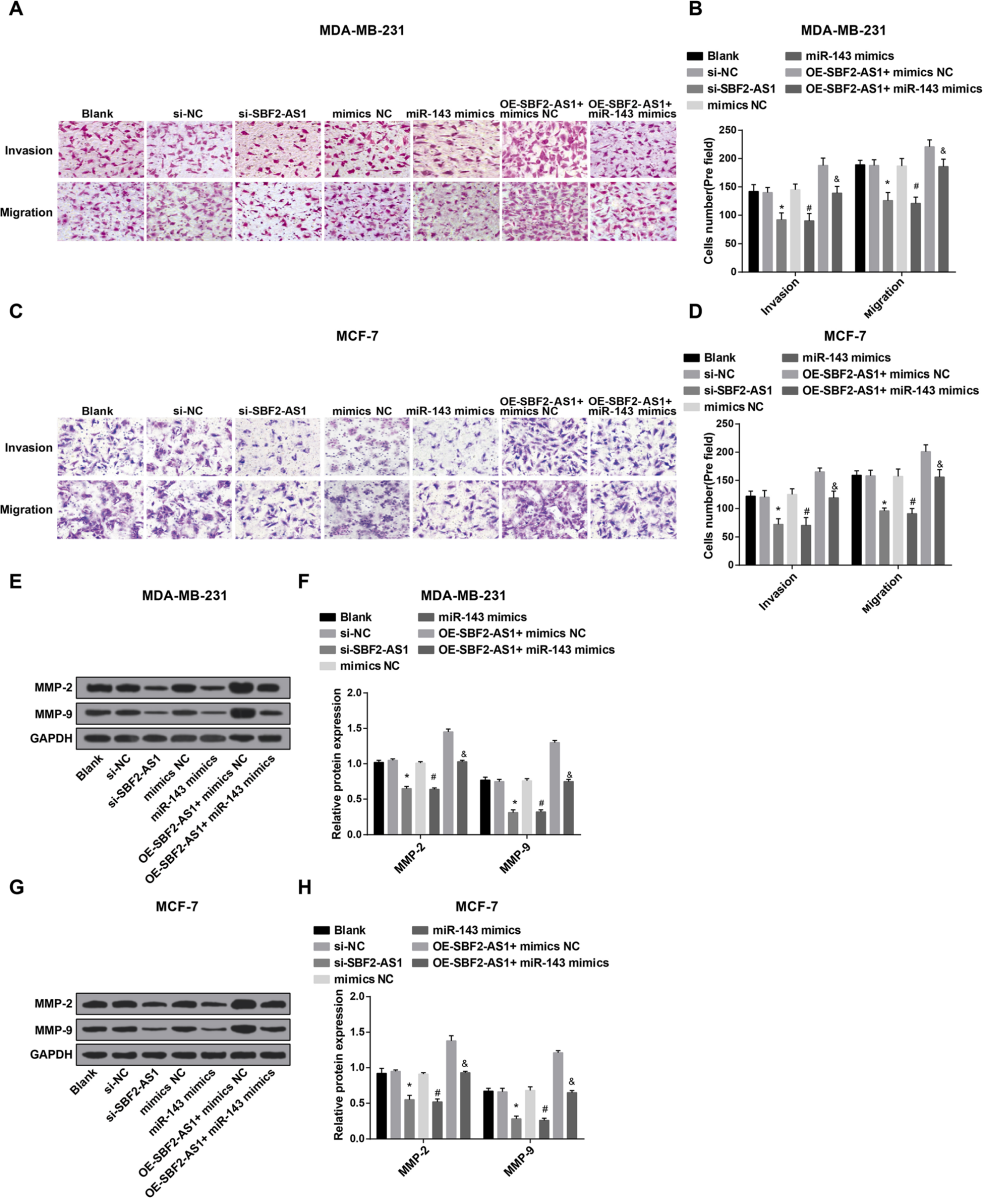
Reduced SBF2-AS1 and overexpressed miR-143 restrain the invasion and migration of BC cells. **a** Invasion and migration abilities of MDA-MB-231 cells were detected by Transwell assay; **b** Statistical results of the number of migrated and invasive MDA-MB-231 cells; **c** Invasion and migration abilities of MCF-7 cells were detected by Transwell assay; **d** Statistical results of the number of migrated and invasive MCF-7 cells; **e** Protein bands of MMP-2 and MMP-9 in MDA-MB-231 cells in Western blot analysis; **f** Statistical results of the protein expression of MMP-2 and MMP-9 in MDA-MB-231 cells; **g** Protein bands of MMP-2 and MMP-9 in MCF-7 cells in Western blot analysis; **h** Statistical results of the protein expression of MMP-2 and MMP-9 in MCF-7 cells. The measurement data conforming to the normal distribution were expressed as mean ± standard deviation. One-way ANOVA was used for comparisons among multiple groups, and LSD-t test was used for pairwise comparisons after one-way ANOVA, *N* = 3, * *P* < 0.05 vs the si-NC group; # *P* < 0.05 vs the mimics NC group; & *P* < 0.05 vs the oe-SBF2-AS1 + mimics NC group

Transwell assay was employed to evaluate the migration and invasion of MDA-MB-231 and MCF-7 cells, the results reflected that (Fig. 6a–d) no apparent difference could be observed in invasion and migration abilities of MDA-MB-231 and MCF-7 cells among the blank group, the si-NC group, the mimics NC group and the oe-SBF2-AS1 + miR-143 mimics group (all *P* > 0.05); contrasted to the si-NC group and the mimics NC group, the invasion and migration abilities of MDA-MB-231 and MCF-7 cells were inhibited in the si-SBF2-AS1 group and the miR-143 mimics group; in contrast to the oe-SBF2-AS1 + mimics NC group, the invasion and migration abilities of MDA-MB-231 and MCF-7 cells were prevented in the oe-SBF2-AS1 + miR-143 mimics group (all *P* < 0.05).

The protein expression of MMP-2 and MMP-9 in MDA-MB-231 and MCF-7 cells was identified by Western blot analysis, the outcomes unveiled that (Fig. 6e–h) there was no noticeable difference in protein expression of MMP-2 and MMP-9 in MDA-MB-231 and MCF-7 cells among the blank group, the si-NC group, the mimics NC group and the oe-SBF2-AS1 + miR-143 mimics group (all *P* > 0.05); relative to the si-NC group and the mimics NC group, protein expression of MMP-2 and MMP-9 in MDA-MB-231 and MCF-7 cells was reduced in the si-SBF2-AS1 group and the miR-143 mimics group; contrasted to the oe-SBF2-AS1 + mimics NC group, the protein expression of MMP-2 and MMP-9 in MDA-MB-231 and MCF-7 cells was declined in the oe-SBF2-AS1 + miR-143 mimics group (all *P* < 0.05).

### Reduced SBF2-AS1 and overexpressed miR-143 suppress the tumor growth in nude mice with BC

**Fig. 7:**
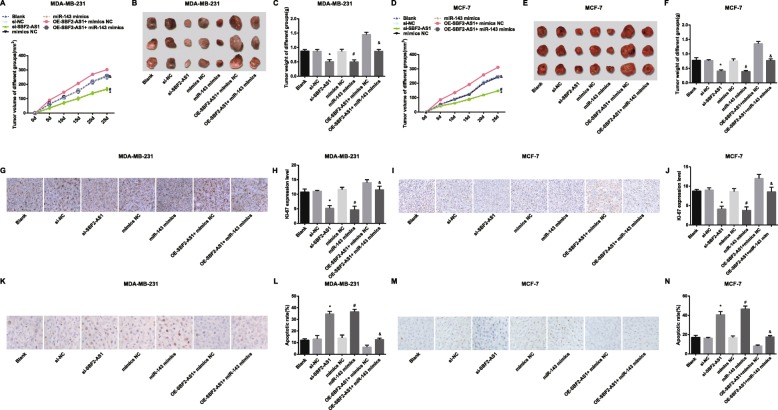
Reduced SBF2-AS1 and overexpressed miR-143 suppress the tumor growth in BC nude mice. **a** The impacts of SBF2-AS1 and miR-143 on MDA-MB-231 tumor growth, the volume of tumor = length × width × height; **b** Representative images of MDA-MB-231 cell tumors in nude mice; **c** Statistical results of the weight of MDA-MB-231 cell tumors in each group; **d** The impacts of SBF2-AS1 and miR-143 on MCF-7 tumor growth, the volume of tumor = length × width × height; **e** Representative images of MCF-7 cell tumors in nude mice; **f** Statistical results of the weight of MCF-7 cell tumors in each group; **g** Ki-67 expression in MDA-MB-231 cell tumors (SABC, × 200); **h** Statistical results of Ki-67 expression in MDA-MB-231 cell tumors; **i** Ki-67 expression in MCF-7 cell tumors (SABC, × 200); **j** Statistical results of Ki-67 expression in MCF-7 cell tumors; **k** Representative images of TUNEL staining in MDA-MB-231 cell tumors (× 400); **l** Apoptosis rate in MDA-MB-231 cell tumors; **m**, Representative images of TUNEL staining in MCF-7 cell tumors (× 400); **n** Apoptosis rate in MCF-7 cell tumors. The measurement data conforming to the normal distribution were expressed as mean ± standard deviation. One-way ANOVA was used for comparisons among multiple groups, and LSD-t test was used for pairwise comparisons after one-way ANOVA, *n* = 6 mice, * *P* < 0.05 vs the si-NC group; # *P* < 0.05 vs the mimics NC group; & *P* < 0.05 vs the oe-SBF2-AS1 + mimics NC group

The impacts of SBF2-AS1 on the tumor growth in BC nude mice were observed by subcutaneous tumorigenesis in nude mice, and the results reflected that (Fig. 7a–f) no evident difference could be found in the weight and volume of the tumors among the blank group, the si-NC group, the mimics NC group and the oe-SBF2-AS1 + miR-143 mimics group (all *P* > 0.05); relative to the si-NC group and the mimics NC group, the weight and volume of the tumors were reduced in the si-SBF2-AS1 group and the miR-143 mimics group; contrasted to the oe-SBF2-AS1 + mimics NC group, the weight and volume of the tumors were restrained in the si-SBF2-AS1 + miR-143 mimics group (all *P* < 0.05), unraveling that the reduced SBF2-AS1 and overexpressed miR-143 could suppress the tumor growth in BC.

Expression of Ki-67 in xenografts was determined by immunohistochemical staining, it came out that (Fig. 7g–j) there were no remarkable difference in Ki-67 expression among the blank group, the si-NC group, the mimics NC group and the oe-SBF2-AS1 + miR-143 mimics group (*P* > 0.05); in contrast to the corresponding NC groups, the expression of Ki-67 was reduced in the si-SBF2-AS1 group and the miR-143 mimics; relative to the oe-SBF2-AS1 + mimics NC group, Ki-67 expression was decreased in the oe-SBF2-AS1 + miR-143 mimics group (all *P* < 0.05).

TUNEL staining was used to evaluate the apoptosis of MDA-MB-231 and MCF-7 cells in xenografts. The results (Fig. 7k–n) mirrored that no significant difference could be observed in apoptosis among the blank group, the si-NC group, the mimics NC group and the oe-SBF2-AS1 + miR-143 mimics group (*P* > 0.05); the apoptosis rate was heightened in the si-SBF2-AS1 group and the miR-143 mimics group, which were respectively compared with their NC groups; in comparison to the oe-SBF2-AS1 + mimics NC group, the apoptosis rate was advanced in the oe-SBF2-AS1 + miR-143 mimics group (all *P* < 0.05).

## Discussion

BC is the most continually diagnosed malignant tumor among women globally, and is the 3rd largest cause of deaths that related to cancers in China [21]. It has been reported that the miRNAs played a significant role of leading molecules in RNA silencing [22], and the critical impacts of lncRNAs on the progressions of multiple complex diseases have been verified [23]. Our research was performed to investigate the roles of lncRNA SBF2-AS1, miR-143, and RRS1 in progression of BC.

We have summarized some results in this study, and one of them showed that SBF2-AS1 was highly expressed in both BC tissues and cells. This ectopic expression of SBF2-AS1 has been revealed by other studies as well. For instance, Chen et al. have unraveled the overexpression of SBF2-AS1 in esophageal squamous cell carcinoma [18], and Zhang et al. have also given out evidence to prove that SBF2-AS1 expression was elevated in both small-cell lung cancer tissues and cell lines [6]. Additionally, we have found that miR-143 was poorly expressed in BC tissues and cells. In accordance with this finding, a recent research has testified that miR-143 was downregulated in patients with BC [24, 25]. Moreover, we have verified that RRS1 was overexpressed in BC patients, and the amplification of RRS1 in BC cell line has also been reported by Song et al. in their study [16].

Furthermore, the inhibitory impacts of reduced SBF2-AS1 and elevated miR-143 with the involvement of inhibited RRS1 on the proliferation of BC cells have been noted in our research. Similar to this outcome, Tian et al. have illuminated that the knockdown of SBF2-AS1 could repress the proliferation ability of acute myeloid leukemia cells in vitro [26], and the suppressive effects of miR-143 on the proliferation of BC cells have been revealed by a previous publication [27]. Additionally, Song et al. have discovered that the degradation of RRS1 has the ability to prevent the proliferation and induce apoptosis and cell cycle arrest of BC cell lines [15]. Interestingly, this result was in line with one of our essential findings that the knockdown of SBF2-AS1 could bind to miR-143 to promote cell cycle arrest and cell apoptosis in BC by repressing RRS1. Similarly, a recent research has suggested that reduced SBF2-AS1 was able to suppress the cell cycle progression of lung adenocarcinoma cells [7], and Zhang et al. have pointed out that the overexpression of miR-143 could induce the apoptosis of bovine granulosa cells [28]. The next outcome in our study reflected that SBF2-AS1 inhibition could upregulate miR-143 to restrain the invasion and migration of BC cells via prohibiting RRS1. The functional role of decreased SBF2-AS1 in cell invasion and migration has been unraveled in colorectal cancer as well [29], and Soheilyfar et al. have clarified that miR-143 could repress the invasion as well as metastasis of BC [30]. What’s more, the similar function of RRS1 knockdown in cervical cancer cells has also been uncovered [31]. Besides, we have found that downregulated SBF2-AS1 and overexpressed miR-143 could decline the growth of BC tumor in vivo. Consistent to our result, it has been revealed that reduced SBF2-AS1 could decelerate the tumor growth of acute myeloid leukemia in vivo [26], and it has been unveiled that overexpressed miR-143 served as a tumor repressor in glioma [32]. Innovatively, the binding relation between SBF2-AS1 and miR-143, as well as the target relation of miR-143 and RRS1 has been verified in this research, which have not been studied before. All of the data were conducive to the investigation process of molecular mechanisms in human diseases.

## Conclusion

In summary, our research demonstrated that the downregulation of lncRNA SBF2-AS1 could inhibit tumorigenesis and progression of BC by sponging miR-143 and repressing RRS1, which may provide novel targets for the management of BC. Nevertheless, more efforts remain to be done to further elucidate the effects of SBF2-AS1, miR-143 and RRS1 on the progression of BC.

## Data Availability

Not applicable.

## Abbreviations

3’UTR: 3′-untranslated region
ANOVA: Analysis of variance
BC: Breast cancer
ceRNA: Competing endogenous RNA
DMEM: Dulbecco’s modified Eagle medium
FBS: Fetal bovine serum
FISH: PBST phosphate buffered solution with tween
GAPDH: Glyceraldehyde phosphate dehydrogenase
lncRNAs: Long non-coding RNAs
LSD-t: Least significant difference t
miRNAs: MicroRNAs
NC: Negative control
OD: Optical density
oe: Overexpressed
PBS: Phosphate buffered saline
PI: Propidium iodide
RT-qPCR: Reverse transcription quantitative polymerase chain reaction
SBF2-AS1: SET-binding factor 2-antisense RNA1
TNM: Tumor, node and metastasis
