# Correction to: Down-regulated lncRNA SBF2-AS1 inhibits tumorigenesis and progression of breast cancer by sponging microRNA-143 and repressing RRS1

**DOI:** 10.1186/s13046-020-01563-5

**Published:** 2020-04-07

**Authors:** Wenfei Xia, Yun Liu, Teng Cheng, Tao Xu, Menglu Dong, Xiaopeng Hu

**Affiliations:** 1 Department of Breast and Thyroid surgery, Division of General Surgery, Tongji Hospital, Tongji Medical College, Huazhong University of Science and Technology, No. 1095, Jiefang Avenue, Qiaokou District, Wuhan City, Hubei Province 430030 People’s Republic of China; 2 Department of ENT, Tongji Hospital, Tongji Medical College, Huazhong University of Science and Technology, Wuhan City, Hubei Province 430030 People’s Republic of China

**Correction to: J Exp Clin Cancer Res (2020) 39: 18**

**https://doi.org/10.1186/s13046-020-1520-5**

The original publication of this manuscript [1] listed the definition of RRS1 as ‘resistance to ralstonia solanacearum 1’ on four occassions. This was incorrect and RSS1 should instead be defined as ‘Ribosome biogenesis regulatory protein homolog’.

In additional, Table 1 needs to be revised as below.

**Table 1:** Primer sequence

Gene	Primer sequence (5′ → 3′)
miR-143	F: TGAGATGAAGCACTGTAGCTC R: GCGAGCACAGAATTAATACGAC
U6	F: CTCGCTTCGGCAGCACA R: AACGCTTCACGAATTTGCGT
SBF2-AS1	F: GGGATGGACTGACAAAAC R: CATCCACAGATGTACCATG
RRS1	F: CCCTACCGGACACCAGAGTAA R: CCGAAAAGGGGTTGAAACTTCC
GAPDH	F: CCACATCGCTCAGACACCAT R: ACCAGGCGCCCAATACG

The authors apologize for the inconvenience that the corrections caused.
